# Substitution of indium for chromium in TlIn_5−*x*_Cr_*x*_Se_8_: crystal structure of TlIn_4.811(5)_Cr_0.189(5)_Se_8_

**DOI:** 10.1107/S2056989017003292

**Published:** 2017-03-14

**Authors:** Robin Lefèvre, David Berthebaud, Franck Gascoin

**Affiliations:** a Laboratoire CRISMAT UMR6508, 6 Blvd du Maréchal Juin, 14050 Caen Cedex 4, France

**Keywords:** crystal structure, selenide, inter­metallics, pseudo-hollandite, solid solution

## Abstract

The new thallium penta­(indium/chromium) octa­selenide, TlIn_4.811(5)_Cr_0.189(5)_Se_8_, has been synthesized by solid-state reaction. It crystallizes isotypically with TlIn_5_Se_8_ in the space group *C*2/*m*. Although the two Tl positions are disordered and only partially occupied, no Tl deficiency was observed. The insertion of chromium in the structure has been confirmed by EDS analysis. Chromium substitutes indium exclusively at one of three In sites, *viz.* at one of the positions with site symmetry 2/*m* (Wyckoff position 2*a*). In the crystal structure, edge-sharing InSe_6_ octa­hedra, and (In,Cr)Se_6_ octa­hedra and InSe_4_ tetra­hedra make up two types of columns that are linked into a framework in which two different types of channels parallel to [010] are present. The Tl atoms are located in the larger of the channels, whereas the other, smaller channel remains unoccupied.

## Abstract 2

A substitution of indium for chromium in TlIn_5_Se_8_ led to the isostructural solid solution TlIn_4.8_Cr_0.2_Se_8_ with only one (Wyckoff position 2*a*) of three In positions occupied by Cr atoms.

## Chemical context   

This study is part of an on-going project focused on low-dimensional chalcogenides with low thermal conductivity. Quasi one-dimensional networks are of great inter­est for thermoelectric applications. Such structures can combine structural disorder, responsible for scattering of phonons, to an electronically conductive network.

Recently, low thermal conductive compounds belonging to the family of pseudo-hollandites were studied. A thermoelectric figure of merit up to ZT = 0.5 at 800 K was found for TlCr_5_Se_8_ (Takahashi *et al.*, 2013 ▸). This discovery inspired further studies on this class of materials. Pseudo-hollandites are compounds with general formula *AM*_5_ *X*_8_, (*A* = alkali metal, alkaline earth metal, Tl; *M* = V, Ti, Cr; *X* = S, Se, Te), the structures of which are made up from CdI_2_-type layers and double chains of *MX*_6_ octa­hedra sharing edges and faces in such a way that channels are created along one axis in which the *A* cations are located. Monoclinic TlCr_5_Se_8_ and the related triclinic compound Ba_0.5_Cr_5_Se_8_ have thermal conductivities well below 1 W m^−1^ K^−1^ from room temperature to 873 K (Lefèvre *et al.*, 2015 ▸, 2016 ▸). As part of our project, we successfully synthesized a solid solution of TlV_5–*x*_Cr_*x*_Se_8_ (*x* = 0, 1, 2, 3, 4, 5) and studied the magnetism and thermoelectric properties of TlV_5_Se_8_ (Maier *et al.*, 2015 ▸).

Working on a similar compound, monoclinic TlIn_5_Se_8_, we attempted to synthesize a solid solution TlIn_5–*x*_Cr_*x*_Se_8_. Initially, the nominal composition TlIn_4_CrSe_8_ was chosen so that chromium fully substitutes indium at the octa­hedral site (Wyckoff position 2*a*) of TlIn_5_Se_8_. Here we present the structure of one compound of the solid solution series TlIn_5–*x*_Cr_*x*_Se_8_ (*x* = 0.189) with only a partial substitution of indium for chromium at this site.

## Structural commentary   

**Figure 1:**
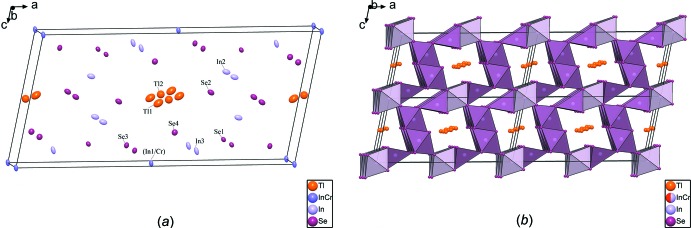
The crystal structure of TlIn_4.811(5)_Cr_0.189(5)_Se_8_. (*a*) Representation by atoms displayed with displacement ellipsoids at the 50% probability level; (*b*) polyhedral representation.

The composition of the crystals as determined from the refinement is in good agreement with the EDS analysis. The refined structure is represented in Fig. 1 ▸, both as individual atoms and in a polyhedral representation. All atoms in the asymmetric unit (two Tl, one mixed-occupied In/Cr, two In and four Se sites) are located on special positions. Except Tl2 and mixed-occupied (In1/Cr) on positions with site symmetry 2/*m* (Wyckoff positions 2*d* and 2*a*, respectively), all other atoms are located on a mirror plane (4*i*).

Indium atoms are found in octa­hedral (In1, In2) and tetra­hedral (In3) environments by selenium atoms. Only one of the indium atoms, In1, shares its position with chromium in an octa­hedral environment. The (In1,Cr)Se_6_ and In2Se_6_ octa­hedra form two types of columns. One column is made up only of edge-sharing In2Se_6_ octa­hedra in a zigzag shape. The second column is made up of alternating (In1/Cr)Se_6_ octa­hedra and In3Se_4_ tetra­hedra connected by edge-sharing, likewise in a zigzag shape. These two building units are linked together to form a framework in which two types of channels propagating parallel to [010] are present. One of the channels hosts the two partly occupied Tl atoms, while the other is smaller and thus empty. Compared to the pseudo-hollandite network, the infinite planes are broken into columns in the title structure, leaving a supplementary channel at the junction of the columns and the double chains.

**Figure 2:**
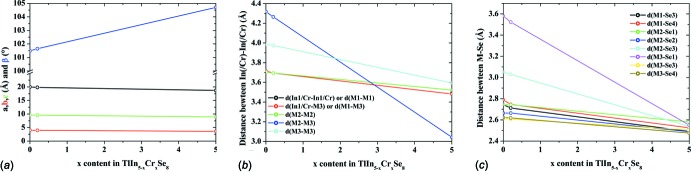
Evolution of (*a*) the unit-cell parameters, (*b*) *M*—*M* distances and (*c*) *M*—Se distances depending on *x* in the TlIn_5–*x*_Cr_*x*_Se_8_ solid solution series.

The existence of the title solid solution is in agreement with the decrease or increase of unit-cell parameters of TlIn_5–*x*_Cr_*x*_Se_8_ from *x* = 0 (Walther & Deiseroth, 1998 ▸) to *x* = 5 (Klepp & Boller, 1983 ▸), as explicited in Fig. 2 ▸ *a*. Further, the decrease in the determined metal-to-metal and metal-to-selenium distances shows a clear trend in agreement with the increase of the chromium content (Fig. 2 ▸ *b*,*c*).

## Synthesis and crystallization   

To prevent oxidation of reactants and products, all mani­p­ulations were performed under inert gas or vacuum (glove box or sealed containers). The elements, Cr (powder, 325 mesh, 99%), In (teardrops, 4 mm, 99.9%) and Se (shots, 99.999%), all from Alfa Aesar, were used as received; Tl (granules, 99.99%), as well from Alfa Aesar, received in water, was first rinsed and dried before being stored in a glovebox. The elements Tl, In, Cr and Se in the molar ratio 1:4:1:8 were loaded directly in a fused silica tube that was subsequently evacuated and flame sealed. The mixture was first heated up to 723 K within 7 h for half a day, and then to 1073 K in 7 h for half a day. The mixture was then cooled down to room temperature in 48 h. An inter­mediate annealing process at 873 K for 15 h was performed. Single crystals were extracted from the bulk.

The bulk sample quality was checked by means of X-ray powder diffraction using a X-Pert Pro Panalytical diffrac­t­ometer (Cu *K*α_1,2_ radiations) equipped with a PIXCEL detector. Phase identification was performed with *X’Pert HighScore plus* (Panalytical, 2009 ▸). Phase analysis using X-ray powder diffraction revealed at first sight TlCr_5_Se_8_ (Klepp & Boller, 1983 ▸) and TlIn_5_Se_8_ (Walther & Deiseroth, 1998 ▸). However, the Bragg positions of these reflections were clearly shifted, pointing to the presence of a TlIn_5–*x*_Cr_*x*_Se_8_ solid solution.

Energy Dispersive X-Ray Spectroscopy (EDS) analyses were also performed to check the composition using a scanning electron microscope (SEM; ZEISS Supra 55, 15 kV). Analysis on basis of nine measured crystallites confirmed the presence of four elements, with a determined average molar composition of Tl 1.05; In 4.54; Cr 0.46; Se 9.1.

## Refinement   

Crystal data, data collection and structure refinement details are summarized in Table 1 ▸.

Structure solution using *SUPERFLIP* (Palatinus & Chapuis, 2007 ▸) led to one thallium site, three indium sites and four selenium sites. Refinement of Tl1 in position (0.5,0,0.5) resulted in large anisotropic displacement parameters. As previously reported, Tl1 usually is located at a partly occupied position around the center position with approximate coordinates of (0.46, 0, 0.52) (Walther & Deiseroth, 1998 ▸). Consideration of the split model (in addition, the In1 site occupancy refined to 0.81 while other indium sites were modelled as fully occupied) led to much more reasonable displacement parameters and improved reliability factors. At that step, the reliability factors were: GOF(all reflections) = 2.42 and *wR* (all reflections) = 0.056, while the maximum and minimum electron densities were +10.28 and −6.03 e^−^ Å^−3^.

The insertion of chromium in the structure has been confirmed by EDS analysis. Consideration of a mixed-occupied In/Cr site for the original In1 position (same coordinates and anisotropic displacement parameters for the two atoms and full occupation for this site) led to a further improvement of reliability factors [GOF (all) = 2.06 and *wR*(all) = 0.0476] and a decrease of the residual electron densities to +9.71 and −5.83 e^−^ Å^−3^. The maximum electron density was found on position (0, 0.5, 0.5). This position is between the partially occupied Tl1 atoms. Thus, a second partially occupied thallium atom, Tl2, was introduced. The two thallium sites are non-simultaneously occupied. The displacement parameter of Tl2 was modelled as isotropic due to its low occupancy compared to Tl1. Adding this second Tl site significantly reduced the residual electron density to final values of +1.41 and −1.69 e^−^ Å^−3^. These density peaks are found at 0.82 and 0.73 Å, respectively, from atoms Se2 and In3.

## Supplementary Material

Crystal structure: contains datablock(s) I. DOI: 10.1107/S2056989017003292/wm5368sup1.cif

Structure factors: contains datablock(s) I. DOI: 10.1107/S2056989017003292/wm5368Isup2.hkl

CCDC reference: 1535178

Additional supporting information:  crystallographic information; 3D view; checkCIF report

## Figures and Tables

**Table 1:** Experimental details

Crystal data	
Chemical formula	TlIn_4.811_Cr_0.189_Se_8_
*M*_r_	1398.3
Crystal system, space group	Monoclinic, *C*2/*m*
Temperature (K)	293
*a*, *b*, *c* (Å)	19.8257 (18), 3.9754 (4), 9.5881 (9)
β (°)	101.645 (5)
*V* (Å^3^)	740.13 (12)
*Z*	2
Radiation type	Mo *K*α
μ (mm^−1^)	37.98
Crystal size (mm)	0.13 × 0.11 × 0.10

Data collection	
Diffractometer	Bruker APEXII CCD area detector
Absorption correction	Numerical (*SADABS*; Bruker, 2004 ▸)
*T*_min_, *T*_max_	0.405, 0.747
No. of measured, independent and observed [*I* > 3σ(*I*)] reflections	5647, 1537, 1181
*R*_int_	0.028
(sin θ/λ)_max_ (Å^−1^)	0.770

Refinement	
*R*[*F* > 3σ(*F*)], *wR*(*F*), *S*	0.030, 0.031, 1.34
No. of reflections	1537
No. of parameters	50
Δρ_max_, Δρ_min_ (e Å^−3^)	1.41, −1.69

Computer programs: *APEX2* and *SAINT* (Bruker, 2004 ▸), *SUPERFLIP* (Palatinus & Chapuis, 2007 ▸), *DIAMOND* (Brandenburg & Putz, 2014 ▸), *JANA2006* (Petříček *et al.*, 2014 ▸) and *publCIF* (Westrip, 2010 ▸).

## supplementary crystallographic information

### Crystal data

**Table:**

TlIn_4.811_Cr_0.189_Se_8_	*F*(000) = 1187
*M**_r_* = 1398.3	*D*_x_ = 6.274 Mg m^−^^3^
Monoclinic, *C*2/*m*	Mo *K*α radiation, λ = 0.71073 Å
Hall symbol: -C 2y	Cell parameters from 2692 reflections
*a* = 19.8257 (18) Å	θ = 2.7–32.8°
*b* = 3.9754 (4) Å	µ = 37.98 mm^−^^1^
*c* = 9.5881 (9) Å	*T* = 293 K
β = 101.645 (5)°	Irregular, black
*V* = 740.13 (12) Å^3^	0.13 × 0.11 × 0.10 mm
*Z* = 2	

### Data collection

**Table:**

Bruker APEXII CCD area detector diffractometer	1537 independent reflections
Radiation source: X-ray tube	1181 reflections with *I* > 3σ(*I*)
Graphite monochromator	*R*_int_ = 0.028
Detector resolution: 8.3333 pixels mm^-1^	θ_max_ = 33.2°, θ_min_ = 2.1°
ω and φ scans	*h* = −30→26
Absorption correction: numerical (*SADABS*; Bruker, 2004)	*k* = −5→6
*T*_min_ = 0.405, *T*_max_ = 0.747	*l* = −12→14
5647 measured reflections	

### Refinement

**Table:**

Refinement on *F*	0 restraints
*R*[*F* > 3σ(*F*)] = 0.030	4 constraints
*wR*(*F*) = 0.031	Weighting scheme based on measured s.u.'s *w* = 1/(σ^2^(*F*) + 0.0001*F*^2^)
*S* = 1.34	(Δ/σ)_max_ = 0.003
1537 reflections	Δρ_max_ = 1.41 e Å^−^^3^
50 parameters	Δρ_min_ = −1.69 e Å^−^^3^

### Fractional atomic coordinates and isotropic or equivalent isotropic displacement parameters (Å^2^)

**Table:**

	*x*	*y*	*z*	*U*_iso_*/*U*_eq_	Occ. (<1)
Tl1	0.46491 (5)	0	0.52551 (8)	0.0407 (3)	0.4631 (16)
Tl2	0	0.5	0.5	0.032 (2)*	0.074 (3)
In1	0	0	0	0.0155 (2)	0.811 (5)
Cr	0	0	0	0.0155 (2)	0.189 (5)
In2	0.28034 (3)	0	0.66298 (5)	0.02131 (15)	
In3	0.36250 (2)	0	0.10972 (5)	0.02052 (15)	
Se1	0.24067 (3)	0	0.15323 (6)	0.01295 (17)	
Se2	0.16279 (4)	0	0.47058 (7)	0.0197 (2)	
Se3	0.58556 (3)	0	0.11945 (6)	0.01640 (19)	
Se4	0.06189 (3)	0	0.76833 (7)	0.01829 (19)	

### Atomic displacement parameters (Å^2^)

**Table:**

	*U*^11^	*U*^22^	*U*^33^	*U*^12^	*U*^13^	*U*^23^
Tl1	0.0434 (6)	0.0352 (4)	0.0376 (4)	0	−0.0058 (3)	0
In1	0.0110 (4)	0.0129 (4)	0.0240 (4)	0	0.0069 (3)	0
Cr	0.0110 (4)	0.0129 (4)	0.0240 (4)	0	0.0069 (3)	0
In2	0.0346 (3)	0.0138 (2)	0.01692 (19)	0	0.00868 (19)	0
In3	0.0158 (2)	0.0166 (2)	0.0327 (3)	0	0.01323 (19)	0
Se1	0.0129 (3)	0.0129 (3)	0.0142 (3)	0	0.0053 (2)	0
Se2	0.0256 (4)	0.0174 (3)	0.0183 (3)	0	0.0094 (2)	0
Se3	0.0121 (3)	0.0179 (3)	0.0187 (3)	0	0.0019 (2)	0
Se4	0.0189 (3)	0.0153 (3)	0.0204 (3)	0	0.0035 (2)	0

### Geometric parameters (Å, º)

**Table:**

Tl1—Tl1^i^	3.9754 (8)	Cr—Se3^xiii^	2.7122 (5)
Tl1—Tl1^ii^	3.9754 (8)	Cr—Se4^xiv^	2.7460 (8)
Tl1—Tl1^iii^	1.5654 (15)	Cr—Se4^viii^	2.7460 (8)
Tl1—Tl2^iv^	0.7827 (11)	In2—In2^i^	3.9754 (8)
Tl1—Se2^v^	3.2249 (11)	In2—In2^ii^	3.9754 (8)
Tl1—Se2^vi^	3.2249 (11)	In2—In2^v^	3.6944 (8)
Tl1—Se3^iii^	3.7369 (13)	In2—In2^vi^	3.6944 (8)
Tl1—Se4^iv^	3.3558 (9)	In2—Se1^v^	2.7433 (6)
Tl1—Se4^vii^	3.3558 (9)	In2—Se1^vi^	2.7433 (6)
Tl1—Se4^v^	3.4010 (9)	In2—Se2	2.6644 (9)
Tl1—Se4^vi^	3.4010 (9)	In2—Se2^v^	2.7286 (7)
Tl2—Tl2^i^	3.9754 (8)	In2—Se2^vi^	2.7286 (7)
Tl2—Tl2^ii^	3.9754 (8)	In2—Se3^iii^	3.0300 (9)
Tl2—Se2	3.8489 (8)	In3—In3^i^	3.9754 (8)
Tl2—Se2^ii^	3.8489 (8)	In3—In3^ii^	3.9754 (8)
Tl2—Se2^viii^	3.8489 (8)	In3—Se1	2.5328 (9)
Tl2—Se2^ix^	3.8489 (8)	In3—Se1^xii^	3.5227 (7)
Tl2—Se4	3.2865 (7)	In3—Se1^xiii^	3.5227 (7)
Tl2—Se4^ii^	3.2865 (7)	In3—Se3^xv^	2.6080 (9)
Tl2—Se4^viii^	3.2865 (7)	In3—Se4^v^	2.6186 (6)
Tl2—Se4^ix^	3.2865 (7)	In3—Se4^vi^	2.6186 (6)
In1—In1^i^	3.9754 (8)	Se1—Se1^i^	3.9754 (8)
In1—In1^ii^	3.9754 (8)	Se1—Se1^ii^	3.9754 (8)
In1—Cr^i^	3.9754 (8)	Se1—Se1^xii^	3.6278 (9)
In1—Cr	0	Se1—Se1^xiii^	3.6278 (9)
In1—Cr^ii^	3.9754 (8)	Se1—Se2	3.6795 (11)
In1—In3^x^	3.6941 (6)	Se1—Se3^x^	3.6207 (9)
In1—In3^xi^	3.6941 (6)	Se1—Se3^xi^	3.6207 (9)
In1—In3^xii^	3.6941 (6)	Se2—Se2^i^	3.9754 (8)
In1—In3^xiii^	3.6941 (6)	Se2—Se2^ii^	3.9754 (8)
In1—Se3^x^	2.7122 (5)	Se2—Se2^v^	3.9294 (10)
In1—Se3^xi^	2.7122 (5)	Se2—Se2^vi^	3.9294 (10)
In1—Se3^xii^	2.7122 (5)	Se2—Se3^x^	3.9432 (9)
In1—Se3^xiii^	2.7122 (5)	Se2—Se3^xi^	3.9432 (9)
In1—Se4^xiv^	2.7460 (8)	Se2—Se4	3.8022 (11)
In1—Se4^viii^	2.7460 (8)	Se3—Se3^i^	3.9754 (8)
Cr—Cr^i^	3.9754 (8)	Se3—Se3^ii^	3.9754 (8)
Cr—Cr^ii^	3.9754 (8)	Se3—Se3^xv^	3.6905 (11)
Cr—In3^x^	3.6941 (6)	Se3—Se4^xvi^	3.8558 (10)
Cr—In3^xi^	3.6941 (6)	Se3—Se4^xvii^	3.8558 (10)
Cr—In3^xii^	3.6941 (6)	Se3—Se4^v^	3.8633 (10)
Cr—In3^xiii^	3.6941 (6)	Se3—Se4^vi^	3.8633 (10)
Cr—Se3^x^	2.7122 (5)	Se4—Se4^i^	3.9754 (8)
Cr—Se3^xi^	2.7122 (5)	Se4—Se4^ii^	3.9754 (8)
Cr—Se3^xii^	2.7122 (5)		
			
Tl1^i^—Tl1—Tl1^ii^	180.0 (5)	In3^xii^—Se1—Se1^xiii^	82.466 (16)
Tl1^i^—Tl1—Tl1^iii^	90.00 (2)	In3^xii^—Se1—Se2	108.174 (19)
Tl1^i^—Tl1—Tl2^iv^	90.00 (2)	In3^xii^—Se1—Se3^x^	42.799 (14)
Tl1^i^—Tl1—Se2^v^	51.949 (18)	In3^xii^—Se1—Se3^xi^	83.442 (16)
Tl1^i^—Tl1—Se2^vi^	128.05 (3)	In3^xiii^—Se1—Se1^i^	124.351 (15)
Tl1^i^—Tl1—Se3^iii^	90.000 (18)	In3^xiii^—Se1—Se1^ii^	55.649 (10)
Tl1^i^—Tl1—Se4^iv^	53.678 (14)	In3^xiii^—Se1—Se1^xii^	82.466 (16)
Tl1^i^—Tl1—Se4^vii^	126.32 (2)	In3^xiii^—Se1—Se1^xiii^	41.459 (14)
Tl1^i^—Tl1—Se4^v^	54.236 (14)	In3^xiii^—Se1—Se2	108.174 (19)
Tl1^i^—Tl1—Se4^vi^	125.76 (2)	In3^xiii^—Se1—Se3^x^	83.442 (16)
Tl1^ii^—Tl1—Tl1^iii^	90.00 (2)	In3^xiii^—Se1—Se3^xi^	42.799 (14)
Tl1^ii^—Tl1—Tl2^iv^	90.00 (2)	Se1^i^—Se1—Se1^xii^	56.776 (11)
Tl1^ii^—Tl1—Se2^v^	128.05 (3)	Se1^i^—Se1—Se1^xiii^	123.224 (17)
Tl1^ii^—Tl1—Se2^vi^	51.949 (18)	Se1^i^—Se1—Se3^x^	56.703 (11)
Tl1^ii^—Tl1—Se3^iii^	90.000 (18)	Se1^i^—Se1—Se3^xi^	123.297 (17)
Tl1^ii^—Tl1—Se4^iv^	126.32 (2)	Se1^ii^—Se1—Se1^xii^	123.224 (17)
Tl1^ii^—Tl1—Se4^vii^	53.678 (14)	Se1^ii^—Se1—Se1^xiii^	56.776 (11)
Tl1^ii^—Tl1—Se4^v^	125.76 (2)	Se1^ii^—Se1—Se3^x^	123.297 (17)
Tl1^ii^—Tl1—Se4^vi^	54.236 (14)	Se1^ii^—Se1—Se3^xi^	56.703 (11)
Tl1^iii^—Tl1—Se2^v^	138.86 (2)	Se1^xii^—Se1—Se1^xiii^	66.448 (14)
Tl1^iii^—Tl1—Se2^vi^	138.86 (2)	Se1^xii^—Se1—Se2	142.961 (13)
Tl1^iii^—Tl1—Se3^iii^	134.67 (5)	Se1^xii^—Se1—Se3^x^	81.396 (15)
Tl1^iii^—Tl1—Se4^iv^	78.22 (4)	Se1^xii^—Se1—Se3^xi^	116.871 (19)
Tl1^iii^—Tl1—Se4^vii^	78.22 (4)	Se1^xiii^—Se1—Se2	142.961 (13)
Tl1^iii^—Tl1—Se4^v^	75.00 (4)	Se1^xiii^—Se1—Se3^x^	116.871 (19)
Tl1^iii^—Tl1—Se4^vi^	75.00 (4)	Se1^xiii^—Se1—Se3^xi^	81.396 (15)
Tl2^iv^—Tl1—Se2^v^	138.86 (3)	Se2—Se1—Se3^x^	65.381 (17)
Tl2^iv^—Tl1—Se2^vi^	138.86 (3)	Se2—Se1—Se3^xi^	65.381 (17)
Tl2^iv^—Tl1—Se3^iii^	134.67 (7)	Se3^x^—Se1—Se3^xi^	66.594 (15)
Tl2^iv^—Tl1—Se4^iv^	78.22 (5)	Tl1^v^—Se2—Tl1^vi^	76.10 (2)
Tl2^iv^—Tl1—Se4^vii^	78.22 (5)	Tl1^v^—Se2—Tl2^i^	7.689 (13)
Tl2^iv^—Tl1—Se4^v^	75.00 (5)	Tl1^v^—Se2—Tl2	69.244 (19)
Tl2^iv^—Tl1—Se4^vi^	75.00 (5)	Tl1^v^—Se2—In2	124.82 (2)
Se2^v^—Tl1—Se2^vi^	76.10 (3)	Tl1^v^—Se2—In2^v^	87.489 (18)
Se2^v^—Tl1—Se3^iii^	68.55 (2)	Tl1^v^—Se2—In2^vi^	148.67 (3)
Se2^v^—Tl1—Se4^iv^	88.171 (18)	Tl1^v^—Se2—Se1	118.10 (2)
Se2^v^—Tl1—Se4^vii^	134.29 (3)	Tl1^v^—Se2—Se2^i^	51.949 (16)
Se2^v^—Tl1—Se4^v^	69.98 (2)	Tl1^v^—Se2—Se2^ii^	128.05 (2)
Se2^v^—Tl1—Se4^vi^	112.22 (3)	Tl1^v^—Se2—Se2^v^	110.899 (15)
Se2^vi^—Tl1—Se3^iii^	68.55 (2)	Tl1^v^—Se2—Se2^vi^	168.61 (2)
Se2^vi^—Tl1—Se4^iv^	134.29 (3)	Tl1^v^—Se2—Se3^x^	61.888 (19)
Se2^vi^—Tl1—Se4^vii^	88.171 (18)	Tl1^v^—Se2—Se3^xi^	98.64 (2)
Se2^vi^—Tl1—Se4^v^	112.22 (3)	Tl1^v^—Se2—Se4	57.184 (19)
Se2^vi^—Tl1—Se4^vi^	69.98 (2)	Tl1^vi^—Se2—Tl2^i^	69.244 (19)
Se3^iii^—Tl1—Se4^iv^	65.75 (2)	Tl1^vi^—Se2—Tl2	7.689 (13)
Se3^iii^—Tl1—Se4^vii^	65.75 (2)	Tl1^vi^—Se2—In2	124.82 (2)
Se3^iii^—Tl1—Se4^v^	136.79 (2)	Tl1^vi^—Se2—In2^v^	148.67 (3)
Se3^iii^—Tl1—Se4^vi^	136.79 (2)	Tl1^vi^—Se2—In2^vi^	87.489 (18)
Se4^iv^—Tl1—Se4^vii^	72.64 (2)	Tl1^vi^—Se2—Se1	118.10 (2)
Se4^iv^—Tl1—Se4^v^	101.558 (16)	Tl1^vi^—Se2—Se2^i^	128.05 (2)
Se4^iv^—Tl1—Se4^vi^	153.22 (4)	Tl1^vi^—Se2—Se2^ii^	51.949 (16)
Se4^vii^—Tl1—Se4^v^	153.22 (4)	Tl1^vi^—Se2—Se2^v^	168.61 (2)
Se4^vii^—Tl1—Se4^vi^	101.558 (16)	Tl1^vi^—Se2—Se2^vi^	110.899 (15)
Se4^v^—Tl1—Se4^vi^	71.529 (19)	Tl1^vi^—Se2—Se3^x^	98.64 (2)
Tl1^xi^—Tl2—Tl1^vi^	180.0 (5)	Tl1^vi^—Se2—Se3^xi^	61.888 (19)
Tl1^xi^—Tl2—Tl2^i^	90	Tl1^vi^—Se2—Se4	57.184 (19)
Tl1^xi^—Tl2—Tl2^ii^	90	Tl2^i^—Se2—Tl2	62.186 (13)
Tl1^xi^—Tl2—Se2	146.55 (2)	Tl2^i^—Se2—In2	125.34 (2)
Tl1^xi^—Tl2—Se2^ii^	146.55 (2)	Tl2^i^—Se2—In2^v^	95.178 (13)
Tl1^xi^—Tl2—Se2^viii^	33.45 (2)	Tl2^i^—Se2—In2^vi^	147.42 (2)
Tl1^xi^—Tl2—Se2^ix^	33.45 (2)	Tl2^i^—Se2—Se1	123.962 (15)
Tl1^xi^—Tl2—Se4	88.30 (5)	Tl2^i^—Se2—Se2^i^	58.907 (12)
Tl1^xi^—Tl2—Se4^ii^	88.30 (5)	Tl2^i^—Se2—Se2^ii^	121.093 (19)
Tl1^xi^—Tl2—Se4^viii^	91.70 (5)	Tl2^i^—Se2—Se2^v^	117.058 (10)
Tl1^xi^—Tl2—Se4^ix^	91.70 (5)	Tl2^i^—Se2—Se2^vi^	167.79 (2)
Tl1^vi^—Tl2—Tl2^i^	90	Tl2^i^—Se2—Se3^x^	67.384 (13)
Tl1^vi^—Tl2—Tl2^ii^	90	Tl2^i^—Se2—Se3^xi^	97.822 (17)
Tl1^vi^—Tl2—Se2	33.45 (2)	Tl2^i^—Se2—Se4	50.873 (12)
Tl1^vi^—Tl2—Se2^ii^	33.45 (2)	Tl2—Se2—In2	125.34 (2)
Tl1^vi^—Tl2—Se2^viii^	146.55 (2)	Tl2—Se2—In2^v^	147.42 (2)
Tl1^vi^—Tl2—Se2^ix^	146.55 (2)	Tl2—Se2—In2^vi^	95.178 (13)
Tl1^vi^—Tl2—Se4	91.70 (5)	Tl2—Se2—Se1	123.962 (15)
Tl1^vi^—Tl2—Se4^ii^	91.70 (5)	Tl2—Se2—Se2^i^	121.093 (19)
Tl1^vi^—Tl2—Se4^viii^	88.30 (5)	Tl2—Se2—Se2^ii^	58.907 (12)
Tl1^vi^—Tl2—Se4^ix^	88.30 (5)	Tl2—Se2—Se2^v^	167.79 (2)
Tl2^i^—Tl2—Tl2^ii^	180.0 (5)	Tl2—Se2—Se2^vi^	117.058 (10)
Tl2^i^—Tl2—Se2	58.907 (6)	Tl2—Se2—Se3^x^	97.822 (17)
Tl2^i^—Tl2—Se2^ii^	121.093 (6)	Tl2—Se2—Se3^xi^	67.384 (13)
Tl2^i^—Tl2—Se2^viii^	58.907 (6)	Tl2—Se2—Se4	50.873 (12)
Tl2^i^—Tl2—Se2^ix^	121.093 (6)	In2—Se2—In2^v^	86.47 (2)
Tl2^i^—Tl2—Se4	52.785 (7)	In2—Se2—In2^vi^	86.47 (2)
Tl2^i^—Tl2—Se4^ii^	127.215 (7)	In2—Se2—Se1	96.79 (3)
Tl2^i^—Tl2—Se4^viii^	52.785 (7)	In2—Se2—Se2^v^	43.875 (14)
Tl2^i^—Tl2—Se4^ix^	127.215 (7)	In2—Se2—Se2^vi^	43.875 (14)
Tl2^ii^—Tl2—Se2	121.093 (6)	In2—Se2—Se3^x^	136.53 (2)
Tl2^ii^—Tl2—Se2^ii^	58.907 (6)	In2—Se2—Se3^xi^	136.53 (2)
Tl2^ii^—Tl2—Se2^viii^	121.093 (6)	In2—Se2—Se4	89.96 (2)
Tl2^ii^—Tl2—Se2^ix^	58.907 (6)	In2^v^—Se2—In2^vi^	93.52 (3)
Tl2^ii^—Tl2—Se4	127.215 (7)	In2^v^—Se2—Se1	47.915 (14)
Tl2^ii^—Tl2—Se4^ii^	52.785 (7)	In2^v^—Se2—Se2^i^	43.241 (14)
Tl2^ii^—Tl2—Se4^viii^	127.215 (7)	In2^v^—Se2—Se2^ii^	136.76 (3)
Tl2^ii^—Tl2—Se4^ix^	52.785 (7)	In2^v^—Se2—Se2^v^	42.593 (15)
Se2—Tl2—Se2^ii^	62.186 (10)	In2^v^—Se2—Se2^vi^	90.05 (2)
Se2—Tl2—Se2^viii^	117.814 (10)	In2^v^—Se2—Se3^x^	50.066 (15)
Se2—Tl2—Se2^ix^	180.0 (5)	In2^v^—Se2—Se3^xi^	95.31 (2)
Se2—Tl2—Se4	63.828 (16)	In2^v^—Se2—Se4	133.020 (14)
Se2—Tl2—Se4^ii^	100.580 (14)	In2^vi^—Se2—Se1	47.915 (14)
Se2—Tl2—Se4^viii^	79.420 (14)	In2^vi^—Se2—Se2^i^	136.76 (3)
Se2—Tl2—Se4^ix^	116.172 (16)	In2^vi^—Se2—Se2^ii^	43.241 (14)
Se2^ii^—Tl2—Se2^viii^	180.0 (5)	In2^vi^—Se2—Se2^v^	90.05 (2)
Se2^ii^—Tl2—Se2^ix^	117.814 (10)	In2^vi^—Se2—Se2^vi^	42.593 (15)
Se2^ii^—Tl2—Se4	100.580 (14)	In2^vi^—Se2—Se3^x^	95.31 (2)
Se2^ii^—Tl2—Se4^ii^	63.828 (16)	In2^vi^—Se2—Se3^xi^	50.066 (15)
Se2^ii^—Tl2—Se4^viii^	116.172 (16)	In2^vi^—Se2—Se4	133.020 (14)
Se2^ii^—Tl2—Se4^ix^	79.420 (14)	Se1—Se2—Se2^v^	67.339 (17)
Se2^viii^—Tl2—Se2^ix^	62.186 (10)	Se1—Se2—Se2^vi^	67.339 (17)
Se2^viii^—Tl2—Se4	79.420 (14)	Se1—Se2—Se3^x^	56.591 (16)
Se2^viii^—Tl2—Se4^ii^	116.172 (16)	Se1—Se2—Se3^xi^	56.591 (16)
Se2^viii^—Tl2—Se4^viii^	63.828 (16)	Se1—Se2—Se4	173.25 (2)
Se2^viii^—Tl2—Se4^ix^	100.580 (14)	Se2^i^—Se2—Se2^ii^	180.0 (5)
Se2^ix^—Tl2—Se4	116.172 (16)	Se2^i^—Se2—Se2^v^	59.612 (13)
Se2^ix^—Tl2—Se4^ii^	79.420 (14)	Se2^i^—Se2—Se2^vi^	120.388 (19)
Se2^ix^—Tl2—Se4^viii^	100.580 (14)	Se2^i^—Se2—Se3^x^	59.729 (11)
Se2^ix^—Tl2—Se4^ix^	63.828 (16)	Se2^i^—Se2—Se3^xi^	120.271 (16)
Se4—Tl2—Se4^ii^	74.429 (12)	Se2^ii^—Se2—Se2^v^	120.388 (19)
Se4—Tl2—Se4^viii^	105.571 (12)	Se2^ii^—Se2—Se2^vi^	59.612 (13)
Se4—Tl2—Se4^ix^	180.0 (5)	Se2^ii^—Se2—Se3^x^	120.271 (16)
Se4^ii^—Tl2—Se4^viii^	180.0 (5)	Se2^ii^—Se2—Se3^xi^	59.729 (11)
Se4^ii^—Tl2—Se4^ix^	105.571 (12)	Se2^v^—Se2—Se2^vi^	60.777 (14)
Se4^viii^—Tl2—Se4^ix^	74.429 (12)	Se2^v^—Se2—Se3^x^	92.659 (16)
In1^i^—In1—In1^ii^	180.0 (5)	Se2^v^—Se2—Se3^xi^	123.81 (2)
In1^i^—In1—In3^x^	57.447 (5)	Se2^v^—Se2—Se4	118.249 (18)
In1^i^—In1—In3^xi^	122.553 (5)	Se2^vi^—Se2—Se3^x^	123.81 (2)
In1^i^—In1—In3^xii^	57.447 (5)	Se2^vi^—Se2—Se3^xi^	92.659 (16)
In1^i^—In1—In3^xiii^	122.553 (5)	Se2^vi^—Se2—Se4	118.249 (18)
In1^i^—In1—Se3^x^	42.872 (10)	Se3^x^—Se2—Se3^xi^	60.542 (13)
In1^i^—In1—Se3^xi^	137.128 (10)	Se3^x^—Se2—Se4	117.94 (2)
In1^i^—In1—Se3^xii^	42.872 (10)	Se3^xi^—Se2—Se4	117.94 (2)
In1^i^—In1—Se3^xiii^	137.128 (10)	Tl1^iii^—Se3—In1^iv^	97.18 (2)
In1^i^—In1—Se4^xiv^	90	Tl1^iii^—Se3—In1^vii^	97.18 (2)
In1^i^—In1—Se4^viii^	90	Tl1^iii^—Se3—Cr^iv^	97.18 (2)
In1^ii^—In1—Cr^i^	180.0 (5)	Tl1^iii^—Se3—Cr^vii^	97.18 (2)
In1^ii^—In1—In3^x^	122.553 (5)	Tl1^iii^—Se3—In2^iii^	74.45 (2)
In1^ii^—In1—In3^xi^	57.447 (5)	Tl1^iii^—Se3—In3^xv^	172.46 (3)
In1^ii^—In1—In3^xii^	122.553 (5)	Tl1^iii^—Se3—Se1^iv^	107.29 (2)
In1^ii^—In1—In3^xiii^	57.447 (5)	Tl1^iii^—Se3—Se1^vii^	107.29 (2)
In1^ii^—In1—Se3^x^	137.128 (10)	Tl1^iii^—Se3—Se2^iv^	49.567 (17)
In1^ii^—In1—Se3^xi^	42.872 (10)	Tl1^iii^—Se3—Se2^vii^	49.567 (17)
In1^ii^—In1—Se3^xii^	137.128 (10)	Tl1^iii^—Se3—Se3^xv^	100.59 (3)
In1^ii^—In1—Se3^xiii^	42.872 (10)	Tl1^iii^—Se3—Se4^xvi^	141.961 (17)
In1^ii^—In1—Se4^xiv^	90	Tl1^iii^—Se3—Se4^xvii^	141.961 (17)
In1^ii^—In1—Se4^viii^	90	Tl1^iii^—Se3—Se4^v^	52.372 (17)
Cr^i^—In1—Cr^ii^	180.0 (5)	Tl1^iii^—Se3—Se4^vi^	52.372 (17)
Cr^i^—In1—In3^x^	57.447 (5)	In1^iv^—Se3—In1^vii^	94.256 (19)
Cr^i^—In1—In3^xi^	122.553 (5)	In1^iv^—Se3—Cr^vii^	94.256 (19)
Cr^i^—In1—In3^xii^	57.447 (5)	In1^iv^—Se3—In2^iii^	132.673 (10)
Cr^i^—In1—In3^xiii^	122.553 (5)	In1^iv^—Se3—In3^xv^	87.929 (17)
Cr^i^—In1—Se3^x^	42.872 (10)	In1^iv^—Se3—Se1^iv^	94.758 (12)
Cr^i^—In1—Se3^xi^	137.128 (10)	In1^iv^—Se3—Se1^vii^	152.58 (2)
Cr^i^—In1—Se3^xii^	42.872 (10)	In1^iv^—Se3—Se2^iv^	95.340 (12)
Cr^i^—In1—Se3^xiii^	137.128 (10)	In1^iv^—Se3—Se2^vii^	146.30 (2)
Cr^ii^—In1—In3^x^	122.553 (5)	In1^iv^—Se3—Se3^i^	42.872 (9)
Cr^ii^—In1—In3^xi^	57.447 (5)	In1^iv^—Se3—Se3^ii^	137.128 (19)
Cr^ii^—In1—In3^xii^	122.553 (5)	In1^iv^—Se3—Se3^xv^	47.128 (10)
Cr^ii^—In1—In3^xiii^	57.447 (5)	In1^iv^—Se3—Se4^xvi^	45.411 (12)
Cr^ii^—In1—Se3^x^	137.128 (10)	In1^iv^—Se3—Se4^xvii^	93.072 (18)
Cr^ii^—In1—Se3^xi^	42.872 (10)	In1^iv^—Se3—Se4^v^	45.298 (13)
Cr^ii^—In1—Se3^xii^	137.128 (10)	In1^iv^—Se3—Se4^vi^	92.907 (19)
Cr^ii^—In1—Se3^xiii^	42.872 (10)	In1^vii^—Se3—Cr^iv^	94.256 (19)
In3^x^—In1—In3^xi^	65.105 (9)	In1^vii^—Se3—Cr^vii^	0.0 (5)
In3^x^—In1—In3^xii^	114.895 (9)	In1^vii^—Se3—In2^iii^	132.673 (10)
In3^x^—In1—In3^xiii^	180.0 (5)	In1^vii^—Se3—In3^xv^	87.929 (17)
In3^x^—In1—Se3^x^	85.411 (15)	In1^vii^—Se3—Se1^iv^	152.58 (2)
In3^x^—In1—Se3^xi^	135.128 (16)	In1^vii^—Se3—Se1^vii^	94.758 (12)
In3^x^—In1—Se3^xii^	44.872 (16)	In1^vii^—Se3—Se2^iv^	146.30 (2)
In3^x^—In1—Se3^xiii^	94.589 (15)	In1^vii^—Se3—Se2^vii^	95.340 (12)
In3^x^—In1—Se4^xiv^	134.936 (11)	In1^vii^—Se3—Se3^i^	137.128 (19)
In3^x^—In1—Se4^viii^	45.064 (11)	In1^vii^—Se3—Se3^ii^	42.872 (9)
In3^xi^—In1—In3^xii^	180.0 (5)	In1^vii^—Se3—Se3^xv^	47.128 (10)
In3^xi^—In1—In3^xiii^	114.895 (9)	In1^vii^—Se3—Se4^xvi^	93.072 (18)
In3^xi^—In1—Se3^x^	135.128 (16)	In1^vii^—Se3—Se4^xvii^	45.411 (12)
In3^xi^—In1—Se3^xi^	85.411 (15)	In1^vii^—Se3—Se4^v^	92.907 (19)
In3^xi^—In1—Se3^xii^	94.589 (15)	In1^vii^—Se3—Se4^vi^	45.298 (13)
In3^xi^—In1—Se3^xiii^	44.872 (16)	Cr^iv^—Se3—Cr^vii^	94.256 (19)
In3^xi^—In1—Se4^xiv^	134.936 (11)	Cr^iv^—Se3—In2^iii^	132.673 (10)
In3^xi^—In1—Se4^viii^	45.064 (11)	Cr^iv^—Se3—In3^xv^	87.929 (17)
In3^xii^—In1—In3^xiii^	65.105 (9)	Cr^iv^—Se3—Se1^iv^	94.758 (12)
In3^xii^—In1—Se3^x^	44.872 (16)	Cr^iv^—Se3—Se1^vii^	152.58 (2)
In3^xii^—In1—Se3^xi^	94.589 (15)	Cr^iv^—Se3—Se2^iv^	95.340 (12)
In3^xii^—In1—Se3^xii^	85.411 (15)	Cr^iv^—Se3—Se2^vii^	146.30 (2)
In3^xii^—In1—Se3^xiii^	135.128 (16)	Cr^iv^—Se3—Se3^i^	42.872 (9)
In3^xii^—In1—Se4^xiv^	45.064 (11)	Cr^iv^—Se3—Se3^ii^	137.128 (19)
In3^xii^—In1—Se4^viii^	134.936 (11)	Cr^iv^—Se3—Se3^xv^	47.128 (10)
In3^xiii^—In1—Se3^x^	94.589 (15)	Cr^iv^—Se3—Se4^xvi^	45.411 (12)
In3^xiii^—In1—Se3^xi^	44.872 (16)	Cr^iv^—Se3—Se4^xvii^	93.072 (18)
In3^xiii^—In1—Se3^xii^	135.128 (16)	Cr^iv^—Se3—Se4^v^	45.298 (13)
In3^xiii^—In1—Se3^xiii^	85.411 (15)	Cr^iv^—Se3—Se4^vi^	92.907 (19)
In3^xiii^—In1—Se4^xiv^	45.064 (11)	Cr^vii^—Se3—In2^iii^	132.673 (10)
In3^xiii^—In1—Se4^viii^	134.936 (11)	Cr^vii^—Se3—In3^xv^	87.929 (17)
Se3^x^—In1—Se3^xi^	94.256 (15)	Cr^vii^—Se3—Se1^iv^	152.58 (2)
Se3^x^—In1—Se3^xii^	85.744 (15)	Cr^vii^—Se3—Se1^vii^	94.758 (12)
Se3^x^—In1—Se3^xiii^	180.0 (5)	Cr^vii^—Se3—Se2^iv^	146.30 (2)
Se3^x^—In1—Se4^xiv^	89.889 (17)	Cr^vii^—Se3—Se2^vii^	95.340 (12)
Se3^x^—In1—Se4^viii^	90.111 (17)	Cr^vii^—Se3—Se3^i^	137.128 (19)
Se3^xi^—In1—Se3^xii^	180.0 (5)	Cr^vii^—Se3—Se3^ii^	42.872 (9)
Se3^xi^—In1—Se3^xiii^	85.744 (15)	Cr^vii^—Se3—Se3^xv^	47.128 (10)
Se3^xi^—In1—Se4^xiv^	89.889 (17)	Cr^vii^—Se3—Se4^xvi^	93.072 (18)
Se3^xi^—In1—Se4^viii^	90.111 (17)	Cr^vii^—Se3—Se4^xvii^	45.411 (12)
Se3^xii^—In1—Se3^xiii^	94.256 (15)	Cr^vii^—Se3—Se4^v^	92.907 (19)
Se3^xii^—In1—Se4^xiv^	90.111 (17)	Cr^vii^—Se3—Se4^vi^	45.298 (13)
Se3^xii^—In1—Se4^viii^	89.889 (17)	In2^iii^—Se3—In3^xv^	98.01 (3)
Se3^xiii^—In1—Se4^xiv^	90.111 (17)	In2^iii^—Se3—Se1^iv^	47.708 (13)
Se3^xiii^—In1—Se4^viii^	89.889 (17)	In2^iii^—Se3—Se1^vii^	47.708 (13)
In1^i^—Cr—In3^x^	57.447 (5)	In2^iii^—Se3—Se2^iv^	43.670 (13)
In1^i^—Cr—In3^xi^	122.553 (5)	In2^iii^—Se3—Se2^vii^	43.670 (13)
In1^i^—Cr—In3^xii^	57.447 (5)	In2^iii^—Se3—Se3^i^	90.000 (11)
In1^i^—Cr—In3^xiii^	122.553 (5)	In2^iii^—Se3—Se3^ii^	90.000 (11)
In1^i^—Cr—Se3^x^	42.872 (10)	In2^iii^—Se3—Se3^xv^	175.04 (3)
In1^i^—Cr—Se3^xi^	137.128 (10)	In2^iii^—Se3—Se4^xvi^	122.44 (2)
In1^i^—Cr—Se3^xii^	42.872 (10)	In2^iii^—Se3—Se4^xvii^	122.44 (2)
In1^i^—Cr—Se3^xiii^	137.128 (10)	In2^iii^—Se3—Se4^v^	114.61 (2)
In1^ii^—Cr—In3^x^	122.553 (5)	In2^iii^—Se3—Se4^vi^	114.61 (2)
In1^ii^—Cr—In3^xi^	57.447 (5)	In3^xv^—Se3—Se1^iv^	66.595 (18)
In1^ii^—Cr—In3^xii^	122.553 (5)	In3^xv^—Se3—Se1^vii^	66.595 (18)
In1^ii^—Cr—In3^xiii^	57.447 (5)	In3^xv^—Se3—Se2^iv^	124.62 (2)
In1^ii^—Cr—Se3^x^	137.128 (10)	In3^xv^—Se3—Se2^vii^	124.62 (2)
In1^ii^—Cr—Se3^xi^	42.872 (10)	In3^xv^—Se3—Se3^i^	90.000 (14)
In1^ii^—Cr—Se3^xii^	137.128 (10)	In3^xv^—Se3—Se3^ii^	90.000 (14)
In1^ii^—Cr—Se3^xiii^	42.872 (10)	In3^xv^—Se3—Se3^xv^	86.96 (2)
Cr^i^—Cr—Cr^ii^	180.0 (5)	In3^xv^—Se3—Se4^xvi^	42.569 (14)
Cr^i^—Cr—In3^x^	57.447 (5)	In3^xv^—Se3—Se4^xvii^	42.569 (14)
Cr^i^—Cr—In3^xi^	122.553 (5)	In3^xv^—Se3—Se4^v^	133.180 (18)
Cr^i^—Cr—In3^xii^	57.447 (5)	In3^xv^—Se3—Se4^vi^	133.180 (18)
Cr^i^—Cr—In3^xiii^	122.553 (5)	Se1^iv^—Se3—Se1^vii^	66.594 (15)
Cr^i^—Cr—Se3^x^	42.872 (10)	Se1^iv^—Se3—Se2^iv^	58.029 (16)
Cr^i^—Cr—Se3^xi^	137.128 (10)	Se1^iv^—Se3—Se2^vii^	91.374 (18)
Cr^i^—Cr—Se3^xii^	42.872 (10)	Se1^iv^—Se3—Se3^i^	56.703 (12)
Cr^i^—Cr—Se3^xiii^	137.128 (10)	Se1^iv^—Se3—Se3^ii^	123.297 (18)
Cr^ii^—Cr—In3^x^	122.553 (5)	Se1^iv^—Se3—Se3^xv^	135.502 (16)
Cr^ii^—Cr—In3^xi^	57.447 (5)	Se1^iv^—Se3—Se4^xvi^	75.321 (16)
Cr^ii^—Cr—In3^xii^	122.553 (5)	Se1^iv^—Se3—Se4^xvii^	108.22 (2)
Cr^ii^—Cr—In3^xiii^	57.447 (5)	Se1^iv^—Se3—Se4^v^	111.678 (12)
Cr^ii^—Cr—Se3^x^	137.128 (10)	Se1^iv^—Se3—Se4^vi^	159.11 (2)
Cr^ii^—Cr—Se3^xi^	42.872 (10)	Se1^vii^—Se3—Se2^iv^	91.374 (18)
Cr^ii^—Cr—Se3^xii^	137.128 (10)	Se1^vii^—Se3—Se2^vii^	58.029 (16)
Cr^ii^—Cr—Se3^xiii^	42.872 (10)	Se1^vii^—Se3—Se3^i^	123.297 (18)
In3^x^—Cr—In3^xi^	65.105 (9)	Se1^vii^—Se3—Se3^ii^	56.703 (12)
In3^x^—Cr—In3^xii^	114.895 (9)	Se1^vii^—Se3—Se3^xv^	135.502 (16)
In3^x^—Cr—In3^xiii^	180.0 (5)	Se1^vii^—Se3—Se4^xvi^	108.22 (2)
In3^x^—Cr—Se3^x^	85.411 (15)	Se1^vii^—Se3—Se4^xvii^	75.321 (16)
In3^x^—Cr—Se3^xi^	135.128 (16)	Se1^vii^—Se3—Se4^v^	159.11 (2)
In3^x^—Cr—Se3^xii^	44.872 (16)	Se1^vii^—Se3—Se4^vi^	111.678 (12)
In3^x^—Cr—Se3^xiii^	94.589 (15)	Se2^iv^—Se3—Se2^vii^	60.542 (13)
In3^x^—Cr—Se4^xiv^	134.936 (11)	Se2^iv^—Se3—Se3^i^	59.729 (11)
In3^x^—Cr—Se4^viii^	45.064 (11)	Se2^iv^—Se3—Se3^ii^	120.271 (16)
In3^xi^—Cr—In3^xii^	180.0 (5)	Se2^iv^—Se3—Se3^xv^	132.827 (19)
In3^xi^—Cr—In3^xiii^	114.895 (9)	Se2^iv^—Se3—Se4^xvi^	116.325 (11)
In3^xi^—Cr—Se3^x^	135.128 (16)	Se2^iv^—Se3—Se4^xvii^	164.40 (2)
In3^xi^—Cr—Se3^xi^	85.411 (15)	Se2^iv^—Se3—Se4^v^	71.825 (16)
In3^xi^—Cr—Se3^xii^	94.589 (15)	Se2^iv^—Se3—Se4^vi^	101.93 (2)
In3^xi^—Cr—Se3^xiii^	44.872 (16)	Se2^vii^—Se3—Se3^i^	120.271 (16)
In3^xi^—Cr—Se4^xiv^	134.936 (11)	Se2^vii^—Se3—Se3^ii^	59.729 (11)
In3^xi^—Cr—Se4^viii^	45.064 (11)	Se2^vii^—Se3—Se3^xv^	132.827 (19)
In3^xii^—Cr—In3^xiii^	65.105 (9)	Se2^vii^—Se3—Se4^xvi^	164.40 (2)
In3^xii^—Cr—Se3^x^	44.872 (16)	Se2^vii^—Se3—Se4^xvii^	116.325 (11)
In3^xii^—Cr—Se3^xi^	94.589 (15)	Se2^vii^—Se3—Se4^v^	101.93 (2)
In3^xii^—Cr—Se3^xii^	85.411 (15)	Se2^vii^—Se3—Se4^vi^	71.825 (16)
In3^xii^—Cr—Se3^xiii^	135.128 (16)	Se3^i^—Se3—Se3^ii^	180.0 (5)
In3^xii^—Cr—Se4^xiv^	45.064 (11)	Se3^i^—Se3—Se3^xv^	90.000 (12)
In3^xii^—Cr—Se4^viii^	134.936 (11)	Se3^i^—Se3—Se4^xvi^	58.969 (11)
In3^xiii^—Cr—Se3^x^	94.589 (15)	Se3^i^—Se3—Se4^xvii^	121.031 (17)
In3^xiii^—Cr—Se3^xi^	44.872 (16)	Se3^i^—Se3—Se4^v^	59.036 (12)
In3^xiii^—Cr—Se3^xii^	135.128 (16)	Se3^i^—Se3—Se4^vi^	120.964 (19)
In3^xiii^—Cr—Se3^xiii^	85.411 (15)	Se3^ii^—Se3—Se3^xv^	90.000 (12)
In3^xiii^—Cr—Se4^xiv^	45.064 (11)	Se3^ii^—Se3—Se4^xvi^	121.031 (17)
In3^xiii^—Cr—Se4^viii^	134.936 (11)	Se3^ii^—Se3—Se4^xvii^	58.969 (11)
Se3^x^—Cr—Se3^xi^	94.256 (15)	Se3^ii^—Se3—Se4^v^	120.964 (19)
Se3^x^—Cr—Se3^xii^	85.744 (15)	Se3^ii^—Se3—Se4^vi^	59.036 (12)
Se3^x^—Cr—Se3^xiii^	180.0 (5)	Se3^xv^—Se3—Se4^xvi^	61.541 (16)
Se3^x^—Cr—Se4^xiv^	89.889 (17)	Se3^xv^—Se3—Se4^xvii^	61.541 (16)
Se3^x^—Cr—Se4^viii^	90.111 (17)	Se3^xv^—Se3—Se4^v^	61.336 (16)
Se3^xi^—Cr—Se3^xii^	180.0 (5)	Se3^xv^—Se3—Se4^vi^	61.336 (16)
Se3^xi^—Cr—Se3^xiii^	85.744 (15)	Se4^xvi^—Se3—Se4^xvii^	62.063 (14)
Se3^xi^—Cr—Se4^xiv^	89.889 (17)	Se4^xvi^—Se3—Se4^v^	90.709 (15)
Se3^xi^—Cr—Se4^viii^	90.111 (17)	Se4^xvi^—Se3—Se4^vi^	122.88 (2)
Se3^xii^—Cr—Se3^xiii^	94.256 (15)	Se4^xvii^—Se3—Se4^v^	122.88 (2)
Se3^xii^—Cr—Se4^xiv^	90.111 (17)	Se4^xvii^—Se3—Se4^vi^	90.709 (15)
Se3^xii^—Cr—Se4^viii^	89.889 (17)	Se4^v^—Se3—Se4^vi^	61.929 (14)
Se3^xiii^—Cr—Se4^xiv^	90.111 (17)	Tl1^x^—Se4—Tl1^xi^	72.644 (18)
Se3^xiii^—Cr—Se4^viii^	89.889 (17)	Tl1^x^—Se4—Tl1^v^	26.78 (3)
In2^i^—In2—In2^v^	57.450 (8)	Tl1^x^—Se4—Tl1^vi^	78.44 (2)
In2^i^—In2—In2^vi^	122.550 (12)	Tl1^x^—Se4—Tl2^i^	13.482 (18)
In2^i^—In2—Se1^v^	43.568 (12)	Tl1^x^—Se4—Tl2	75.169 (16)
In2^i^—In2—Se1^vi^	136.432 (19)	Tl1^x^—Se4—In1^xviii^	105.91 (3)
In2^i^—In2—Se2^v^	43.241 (14)	Tl1^x^—Se4—Cr^xviii^	105.91 (3)
In2^i^—In2—Se2^vi^	136.76 (2)	Tl1^x^—Se4—In3^v^	93.230 (15)
In2^i^—In2—Se3^iii^	90.000 (9)	Tl1^x^—Se4—In3^vi^	162.86 (3)
In2^ii^—In2—In2^v^	122.550 (12)	Tl1^x^—Se4—Se2	78.09 (2)
In2^ii^—In2—In2^vi^	57.450 (8)	Tl1^x^—Se4—Se3^xix^	104.602 (16)
In2^ii^—In2—Se1^v^	136.432 (19)	Tl1^x^—Se4—Se3^xx^	149.63 (3)
In2^ii^—In2—Se1^vi^	43.568 (12)	Tl1^x^—Se4—Se3^v^	61.88 (2)
In2^ii^—In2—Se2^v^	136.76 (2)	Tl1^x^—Se4—Se3^vi^	97.94 (2)
In2^ii^—In2—Se2^vi^	43.241 (14)	Tl1^x^—Se4—Se4^i^	53.678 (12)
In2^v^—In2—In2^vi^	65.100 (11)	Tl1^x^—Se4—Se4^ii^	126.322 (19)
In2^v^—In2—Se1^v^	95.040 (13)	Tl1^xi^—Se4—Tl1^v^	78.44 (2)
In2^v^—In2—Se1^vi^	150.17 (2)	Tl1^xi^—Se4—Tl1^vi^	26.78 (3)
In2^v^—In2—Se2	47.491 (14)	Tl1^xi^—Se4—Tl2^i^	75.169 (16)
In2^v^—In2—Se2^v^	46.041 (16)	Tl1^xi^—Se4—Tl2	13.482 (18)
In2^v^—In2—Se2^vi^	95.149 (19)	Tl1^xi^—Se4—In1^xviii^	105.91 (3)
In2^v^—In2—Se3^iii^	132.304 (17)	Tl1^xi^—Se4—Cr^xviii^	105.91 (3)
In2^vi^—In2—Se1^v^	150.17 (2)	Tl1^xi^—Se4—In3^v^	162.86 (3)
In2^vi^—In2—Se1^vi^	95.040 (13)	Tl1^xi^—Se4—In3^vi^	93.230 (15)
In2^vi^—In2—Se2	47.491 (14)	Tl1^xi^—Se4—Se2	78.09 (2)
In2^vi^—In2—Se2^v^	95.149 (19)	Tl1^xi^—Se4—Se3^xix^	149.63 (3)
In2^vi^—In2—Se2^vi^	46.041 (16)	Tl1^xi^—Se4—Se3^xx^	104.602 (16)
In2^vi^—In2—Se3^iii^	132.304 (17)	Tl1^xi^—Se4—Se3^v^	97.94 (2)
Se1^v^—In2—Se1^vi^	92.86 (2)	Tl1^xi^—Se4—Se3^vi^	61.88 (2)
Se1^v^—In2—Se2	102.70 (2)	Tl1^xi^—Se4—Se4^i^	126.322 (19)
Se1^v^—In2—Se2^v^	84.510 (16)	Tl1^xi^—Se4—Se4^ii^	53.678 (12)
Se1^v^—In2—Se2^vi^	163.74 (3)	Tl1^v^—Se4—Tl1^vi^	71.529 (18)
Se1^v^—In2—Se3^iii^	77.503 (18)	Tl1^v^—Se4—Tl2^i^	13.300 (18)
Se1^vi^—In2—Se2	102.70 (2)	Tl1^v^—Se4—Tl2	74.560 (17)
Se1^vi^—In2—Se2^v^	163.74 (3)	Tl1^v^—Se4—In1^xviii^	130.51 (2)
Se1^vi^—In2—Se2^vi^	84.510 (16)	Tl1^v^—Se4—Cr^xviii^	130.51 (2)
Se1^vi^—In2—Se3^iii^	77.503 (18)	Tl1^v^—Se4—In3^v^	84.600 (17)
Se2—In2—Se2^v^	93.53 (2)	Tl1^v^—Se4—In3^vi^	142.48 (3)
Se2—In2—Se2^vi^	93.53 (2)	Tl1^v^—Se4—Se2	52.84 (2)
Se2—In2—Se3^iii^	179.70 (3)	Tl1^v^—Se4—Se3^xix^	113.150 (12)
Se2^v^—In2—Se2^vi^	93.52 (2)	Tl1^v^—Se4—Se3^xx^	174.69 (2)
Se2^v^—In2—Se3^iii^	86.26 (2)	Tl1^v^—Se4—Se3^v^	85.96 (2)
Se2^vi^—In2—Se3^iii^	86.26 (2)	Tl1^v^—Se4—Se3^vi^	122.07 (3)
In1^iv^—In3—In1^vii^	65.105 (11)	Tl1^v^—Se4—Se4^i^	54.236 (13)
In1^iv^—In3—Cr^iv^	0.0 (5)	Tl1^v^—Se4—Se4^ii^	125.764 (19)
In1^iv^—In3—Cr^vii^	65.105 (11)	Tl1^vi^—Se4—Tl2^i^	74.560 (17)
In1^iv^—In3—In3^i^	57.447 (8)	Tl1^vi^—Se4—Tl2	13.300 (18)
In1^iv^—In3—In3^ii^	122.553 (14)	Tl1^vi^—Se4—In1^xviii^	130.51 (2)
In1^iv^—In3—Se1	146.097 (7)	Tl1^vi^—Se4—Cr^xviii^	130.51 (2)
In1^iv^—In3—Se1^xii^	81.246 (13)	Tl1^vi^—Se4—In3^v^	142.48 (3)
In1^iv^—In3—Se1^xiii^	117.067 (18)	Tl1^vi^—Se4—In3^vi^	84.600 (17)
In1^iv^—In3—Se3^xv^	47.199 (12)	Tl1^vi^—Se4—Se2	52.84 (2)
In1^iv^—In3—Se4^v^	47.930 (17)	Tl1^vi^—Se4—Se3^xix^	174.69 (2)
In1^iv^—In3—Se4^vi^	98.44 (2)	Tl1^vi^—Se4—Se3^xx^	113.150 (12)
In1^vii^—In3—Cr^iv^	65.105 (11)	Tl1^vi^—Se4—Se3^v^	122.07 (3)
In1^vii^—In3—Cr^vii^	0.0 (5)	Tl1^vi^—Se4—Se3^vi^	85.96 (2)
In1^vii^—In3—In3^i^	122.553 (14)	Tl1^vi^—Se4—Se4^i^	125.764 (19)
In1^vii^—In3—In3^ii^	57.447 (8)	Tl1^vi^—Se4—Se4^ii^	54.236 (13)
In1^vii^—In3—Se1	146.097 (7)	Tl2^i^—Se4—Tl2	74.429 (15)
In1^vii^—In3—Se1^xii^	117.067 (18)	Tl2^i^—Se4—In1^xviii^	118.427 (18)
In1^vii^—In3—Se1^xiii^	81.246 (13)	Tl2^i^—Se4—Cr^xviii^	118.427 (18)
In1^vii^—In3—Se3^xv^	47.199 (12)	Tl2^i^—Se4—In3^v^	88.858 (13)
In1^vii^—In3—Se4^v^	98.44 (2)	Tl2^i^—Se4—In3^vi^	153.93 (3)
In1^vii^—In3—Se4^vi^	47.930 (17)	Tl2^i^—Se4—Se2	65.299 (14)
Cr^iv^—In3—Cr^vii^	65.105 (11)	Tl2^i^—Se4—Se3^xix^	109.398 (10)
Cr^iv^—In3—In3^i^	57.447 (8)	Tl2^i^—Se4—Se3^xx^	162.88 (2)
Cr^iv^—In3—In3^ii^	122.553 (14)	Tl2^i^—Se4—Se3^v^	73.913 (13)
Cr^iv^—In3—Se1	146.097 (7)	Tl2^i^—Se4—Se3^vi^	110.197 (19)
Cr^iv^—In3—Se1^xii^	81.246 (13)	Tl2^i^—Se4—Se4^i^	52.785 (10)
Cr^iv^—In3—Se1^xiii^	117.067 (18)	Tl2^i^—Se4—Se4^ii^	127.215 (17)
Cr^iv^—In3—Se3^xv^	47.199 (12)	Tl2—Se4—In1^xviii^	118.427 (18)
Cr^iv^—In3—Se4^v^	47.930 (17)	Tl2—Se4—Cr^xviii^	118.427 (18)
Cr^iv^—In3—Se4^vi^	98.44 (2)	Tl2—Se4—In3^v^	153.93 (3)
Cr^vii^—In3—In3^i^	122.553 (14)	Tl2—Se4—In3^vi^	88.858 (13)
Cr^vii^—In3—In3^ii^	57.447 (8)	Tl2—Se4—Se2	65.299 (14)
Cr^vii^—In3—Se1	146.097 (7)	Tl2—Se4—Se3^xix^	162.88 (2)
Cr^vii^—In3—Se1^xii^	117.067 (18)	Tl2—Se4—Se3^xx^	109.398 (10)
Cr^vii^—In3—Se1^xiii^	81.246 (13)	Tl2—Se4—Se3^v^	110.197 (19)
Cr^vii^—In3—Se3^xv^	47.199 (12)	Tl2—Se4—Se3^vi^	73.913 (13)
Cr^vii^—In3—Se4^v^	98.44 (2)	Tl2—Se4—Se4^i^	127.215 (17)
Cr^vii^—In3—Se4^vi^	47.930 (17)	Tl2—Se4—Se4^ii^	52.785 (10)
In3^i^—In3—In3^ii^	180.0 (5)	In1^xviii^—Se4—Cr^xviii^	0.0 (5)
In3^i^—In3—Se1^xii^	55.649 (9)	In1^xviii^—Se4—In3^v^	87.01 (2)
In3^i^—In3—Se1^xiii^	124.351 (13)	In1^xviii^—Se4—In3^vi^	87.01 (2)
In3^i^—In3—Se4^v^	40.619 (12)	In1^xviii^—Se4—Se2	174.94 (2)
In3^i^—In3—Se4^vi^	139.381 (18)	In1^xviii^—Se4—Se3^xix^	44.701 (13)
In3^ii^—In3—Se1^xii^	124.351 (13)	In1^xviii^—Se4—Se3^xx^	44.701 (13)
In3^ii^—In3—Se1^xiii^	55.649 (9)	In1^xviii^—Se4—Se3^v^	44.590 (12)
In3^ii^—In3—Se4^v^	139.381 (18)	In1^xviii^—Se4—Se3^vi^	44.590 (12)
In3^ii^—In3—Se4^vi^	40.619 (12)	In1^xviii^—Se4—Se4^i^	90.000 (14)
Se1—In3—Se1^xii^	71.494 (18)	In1^xviii^—Se4—Se4^ii^	90.000 (14)
Se1—In3—Se1^xiii^	71.494 (18)	Cr^xviii^—Se4—In3^v^	87.01 (2)
Se1—In3—Se3^xv^	133.67 (3)	Cr^xviii^—Se4—In3^vi^	87.01 (2)
Se1—In3—Se4^v^	113.92 (2)	Cr^xviii^—Se4—Se2	174.94 (2)
Se1—In3—Se4^vi^	113.92 (2)	Cr^xviii^—Se4—Se3^xix^	44.701 (13)
Se1^xii^—In3—Se1^xiii^	68.701 (13)	Cr^xviii^—Se4—Se3^xx^	44.701 (13)
Se1^xii^—In3—Se3^xv^	70.606 (18)	Cr^xviii^—Se4—Se3^v^	44.590 (12)
Se1^xii^—In3—Se4^v^	95.083 (14)	Cr^xviii^—Se4—Se3^vi^	44.590 (12)
Se1^xii^—In3—Se4^vi^	160.95 (2)	Cr^xviii^—Se4—Se4^i^	90.000 (14)
Se1^xiii^—In3—Se3^xv^	70.606 (18)	Cr^xviii^—Se4—Se4^ii^	90.000 (14)
Se1^xiii^—In3—Se4^v^	160.95 (2)	In3^v^—Se4—In3^vi^	98.76 (2)
Se1^xiii^—In3—Se4^vi^	95.083 (14)	In3^v^—Se4—Se2	89.70 (2)
Se3^xv^—In3—Se4^v^	95.08 (2)	In3^v^—Se4—Se3^xix^	42.356 (17)
Se3^xv^—In3—Se4^vi^	95.08 (2)	In3^v^—Se4—Se3^xx^	92.50 (2)
Se4^v^—In3—Se4^vi^	98.76 (2)	In3^v^—Se4—Se3^v^	83.236 (17)
In2^v^—Se1—In2^vi^	92.86 (2)	In3^v^—Se4—Se3^vi^	131.55 (3)
In2^v^—Se1—In3	112.287 (19)	In3^v^—Se4—Se4^i^	40.619 (12)
In2^v^—Se1—In3^xii^	84.802 (14)	In3^v^—Se4—Se4^ii^	139.38 (2)
In2^v^—Se1—In3^xiii^	136.64 (2)	In3^vi^—Se4—Se2	89.70 (2)
In2^v^—Se1—Se1^i^	43.568 (12)	In3^vi^—Se4—Se3^xix^	92.50 (2)
In2^v^—Se1—Se1^ii^	136.43 (2)	In3^vi^—Se4—Se3^xx^	42.356 (17)
In2^v^—Se1—Se1^xii^	100.184 (12)	In3^vi^—Se4—Se3^v^	131.55 (3)
In2^v^—Se1—Se1^xiii^	166.119 (16)	In3^vi^—Se4—Se3^vi^	83.236 (17)
In2^v^—Se1—Se2	47.575 (13)	In3^vi^—Se4—Se4^i^	139.38 (2)
In2^v^—Se1—Se3^x^	54.788 (16)	In3^vi^—Se4—Se4^ii^	40.619 (12)
In2^v^—Se1—Se3^xi^	102.65 (2)	Se2—Se4—Se3^xix^	131.745 (18)
In2^vi^—Se1—In3	112.287 (19)	Se2—Se4—Se3^xx^	131.745 (18)
In2^vi^—Se1—In3^xii^	136.64 (2)	Se2—Se4—Se3^v^	138.720 (15)
In2^vi^—Se1—In3^xiii^	84.802 (14)	Se2—Se4—Se3^vi^	138.720 (15)
In2^vi^—Se1—Se1^i^	136.43 (2)	Se2—Se4—Se4^i^	90.000 (14)
In2^vi^—Se1—Se1^ii^	43.568 (12)	Se2—Se4—Se4^ii^	90.000 (14)
In2^vi^—Se1—Se1^xii^	166.119 (16)	Se3^xix^—Se4—Se3^xx^	62.063 (14)
In2^vi^—Se1—Se1^xiii^	100.184 (12)	Se3^xix^—Se4—Se3^v^	57.123 (15)
In2^vi^—Se1—Se2	47.575 (13)	Se3^xix^—Se4—Se3^vi^	89.291 (19)
In2^vi^—Se1—Se3^x^	102.65 (2)	Se3^xix^—Se4—Se4^i^	58.969 (12)
In2^vi^—Se1—Se3^xi^	54.788 (16)	Se3^xix^—Se4—Se4^ii^	121.031 (18)
In3—Se1—In3^xii^	108.51 (2)	Se3^xx^—Se4—Se3^v^	89.291 (19)
In3—Se1—In3^xiii^	108.51 (2)	Se3^xx^—Se4—Se3^vi^	57.123 (15)
In3—Se1—Se1^xiii^	67.047 (18)	Se3^xx^—Se4—Se4^i^	121.031 (18)
In3—Se1—Se2	135.19 (2)	Se3^xx^—Se4—Se4^ii^	58.969 (12)
In3—Se1—Se3^x^	143.728 (13)	Se3^v^—Se4—Se3^vi^	61.929 (14)
In3—Se1—Se3^xi^	143.728 (13)	Se3^v^—Se4—Se4^i^	59.036 (13)
In3^xii^—Se1—In3^xiii^	68.701 (13)	Se3^v^—Se4—Se4^ii^	120.964 (19)
In3^xii^—Se1—Se1^i^	55.649 (10)	Se3^vi^—Se4—Se4^i^	120.964 (19)
In3^xii^—Se1—Se1^ii^	124.351 (15)	Se3^vi^—Se4—Se4^ii^	59.036 (13)
In3^xii^—Se1—Se1^xii^	41.459 (14)	Se4^i^—Se4—Se4^ii^	180.0 (5)

Symmetry codes: (i) *x*, *y*−1, *z*; (ii) *x*, *y*+1, *z*; (iii) −*x*+1, *y*, −*z*+1; (iv) *x*+1/2, *y*−1/2, *z*; (v) −*x*+1/2, *y*−1/2, −*z*+1; (vi) −*x*+1/2, *y*+1/2, −*z*+1; (vii) *x*+1/2, *y*+1/2, *z*; (viii) −*x*, *y*, −*z*+1; (ix) −*x*, *y*+1, −*z*+1; (x) *x*−1/2, *y*−1/2, *z*; (xi) *x*−1/2, *y*+1/2, *z*; (xii) −*x*+1/2, *y*−1/2, −*z*; (xiii) −*x*+1/2, *y*+1/2, −*z*; (xiv) *x*, *y*, *z*−1; (xv) −*x*+1, *y*, −*z*; (xvi) *x*+1/2, *y*−1/2, *z*−1; (xvii) *x*+1/2, *y*+1/2, *z*−1; (xviii) *x*, *y*, *z*+1; (xix) *x*−1/2, *y*−1/2, *z*+1; (xx) *x*−1/2, *y*+1/2, *z*+1.
